# Invasion of the Red Seaweed *Heterosiphonia japonica* Spans Biogeographic Provinces in the Western North Atlantic Ocean

**DOI:** 10.1371/journal.pone.0062261

**Published:** 2013-04-24

**Authors:** Christine Newton, Matthew E. S. Bracken, Megan McConville, Katherine Rodrigue, Carol S. Thornber

**Affiliations:** 1 Marine Science Center, Northeastern University, Nahant, Massachusetts, United States of America; 2 Department of Biological Sciences, University of Rhode Island, Kingston, Rhode Island, United States of America; University of Connecticut, United States of America

## Abstract

The recent invasion of the red alga *Heterosiphonia japonica* in the western North Atlantic Ocean has provided a unique opportunity to study invasion dynamics across a biogeographical barrier. Native to the western North Pacific Ocean, initial collections in 2007 and 2009 restricted the western North Atlantic range of this invader to Rhode Island, USA. However, through subtidal community surveys, we document the presence of *Heterosiphonia* in coastal waters from Maine to New York, USA, a distance of more than 700 km. This geographical distribution spans a well-known biogeographical barrier at Cape Cod, Massachusetts. Despite significant differences in subtidal community structure north and south of Cape Cod, *Heterosiphonia* was found at all but two sites surveyed in both biogeographic provinces, suggesting that this invader is capable of rapid expansion over broad geographic ranges. Across all sites surveyed, *Heterosiphonia* comprised 14% of the subtidal benthic community. However, average abundances of nearly 80% were found at some locations. As a drifting macrophyte, *Heterosiphonia* was found as intertidal wrack in abundances of up to 65% of the biomass washed up along beaches surveyed. Our surveys suggest that the high abundance of *Heterosiphonia* has already led to marked changes in subtidal community structure; we found significantly lower species richness in recipient communities with higher *Heterosiphona* abundances. Based on temperature and salinity tolerances of the European populations, we believe *Heterosiphonia* has the potential to invade and alter subtidal communities from Florida to Newfoundland in the western North Atlantic.

## Introduction

Non-native species invasions have become a primary focus of research on global change in the past decade [1], [2]. The importance of marine invasions is highlighted by the significant economic and ecological impacts often associated with these species following a successful invasion. While only a small fraction of all introduced species can successfully thrive in a new habitat, their impacts can be dramatic [3], [4]. Economically, invasive marine species have been responsible for the collapse of fisheries and losses in aquaculture, tourism, and marine infrastructure [5]. Invaders may also have substantial ecological impacts by modifying the habitat in which they invade, displacing native species, and altering food webs and community structure [6]. Additionally, marine invasive species have been identified as a major threat to biodiversity [1], [5], [7].

The invasive red seaweed *Heterosiphonia japonica* Yendo (hereafter *Heterosiphonia*), recently discovered in the western North Atlantic Ocean, poses a threat to native biodiversity and ecosystem functioning. First reported in Rhode Island waters in 2007, this species is morphologically and genetically identical to invasive populations of *Heterosiphonia* in the eastern North Atlantic [8], [9]. *Heterosiphonia* was first recorded in France in 1984 and has since become widespread along European coastlines [10]. While the exact vector of introduction to the western North Atlantic is unknown, this species was likely introduced from Europe via ballast water early in the new century [8].

In contrast to its European invaded range [10], [11], [12], *Heterosiphonia* is not particularly abundant in its native range in the western North Pacific Ocean, comprising less than 1% of the macroalgal biomass and only occurring sporadically throughout the year [13], [14]. In both its native and invaded ranges, *Heterosiphonia* occupies shallow, subtidal habitats and is present either on rocky substrata or epiphytic on other macroalgal species, although it has also been found in sandy and soft sediment habitats ([11], [13], C. Newton, pers. obs.).

Initial reports limited the western Atlantic distribution of this species to Rhode Island [8]. However, reports from the invasion of *Heterosiphonia* in Europe suggest that the species is capable of rapid dispersal associated with broad thermal and salinity tolerances and high fecundity due to vegetative propagation of fragmented pseudolaterals [15], [16]. This has led to a wide geographic distribution in Europe, with reports of the invasive alga from Norway to Italy [10]. Based on thermal tolerances across its European range, *Heterosiphonia* has the potential to invade western Atlantic waters from Newfoundland through Florida [16].

Based on these predictions from the eastern Atlantic invasion of *Heterosiphonia*, we sought to: (1) ascertain the present invaded range and relative abundance of *Heterosiphonia* in western Atlantic coastal waters; (2) determine the biological attributes that are associated with recipient communities; (3) establish whether more diverse communities have higher resistance to *Heterosiphonia* invasion; and (4) assess the proportion of drift *Heterosiphonia* in wrack mats washed ashore. We addressed these goals by surveying shallow subtidal communities and adjacent beaches along the northwestern Atlantic shoreline from New York to Maine, USA.

## Methods

**Figure 1:**
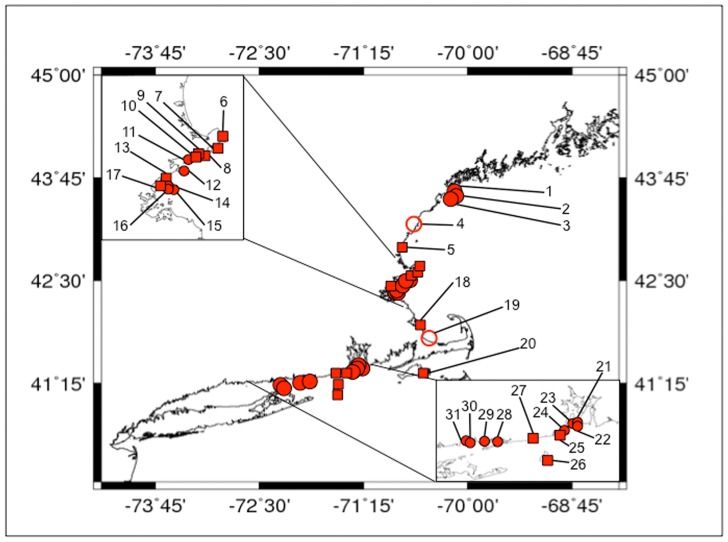
Presence of *Heterosiphonia japonica* in the Western Atlantic Ocean. Numbers correspond to locations listed in Table 1 where *Heterosiphonia* was found in SCUBA, shallow subtidal, and intertidal surveys. Circles indicate locations of *in situ* SCUBA surveys, with filled circles corresponding to locations where *Heterosiphonia* was found and open circles indicating locations where *Heterosiphonia* was absent. Squares correspond to locations where *Heterosiphoina* was found drifting in the shallow subtidal or as intertidal wrack.

We conducted subtidal community surveys at 19 sites between Cape Elizabeth, Maine (43^°^37′N, 70°12′W) and Waterford, Connecticut (41°17′N, 72°09′W), including Southold, New York (41°18′N, 71°55′W), during the summer of 2012 (Fig. 1). This geographic range covers over 700 km of coastline while spanning a well-known biogeographic barrier in the western Atlantic Ocean; Cape Cod, Massachusetts, separates the Acadian biogeographic province from the more southerly Virginian province. These two provinces are characterized by marked differences in water temperature and community structure [17], [18]. Cape Cod is a well-known southern limit for many cold water marine species, as the southward flowing Labrador Current brings down cooler waters, before swinging east along the arm of Cape Cod, and finally returning in a northeasterly direction. However, waters south of Cape Cod are more influenced by the warmer Gulf Stream, particularly during the summer months [17].

Surveys were conducted in both biogeographic provinces between 0 and 6.25 m depth using SCUBA, as *Heterosiphonia* is most commonly found within this depth range. Sites were chosen to include a variable range of exposure, from locations exposed to ocean swells to protected bays.

We also conducted weekly surveys of intertidal wrack mats at five sites in the northern range of our surveys from 28 June 2012 through 02 August 2012 (Table 1). These sites were chosen to encompass a variety of exposures and local topographies. Surveys were conducted at low tide each week. Similar to our subtidal surveys, a 20 m transect was laid parallel to the mean-low water line at each site and a 0.0625 m^2^ quadrat was used to sample every 2 m along the transect. The contents of each quadrat were collected into individual bags and immediately returned to the laboratory where any *Heterosiphonia* present in the quadrat was sorted out. The wet weight of all *Heterosiphonia* and other remaining macrophytes present was recorded after being spun in a salad spinner 15X to remove excess moisture [6].

**Table 1:** Locations of *Heterosiphonia japonica* surveys.

Location	Site Number	Latitude ^o^N	Longitude ^o^W	% cover of *Heterosiphonia*(mean ± S.E.)
**Maine**				
Fort Williams State Park, Cape Elizabeth	1	43°37′31.15″	70°12′46.73″	0.28±0.28
Two Lights State Park, Cape Elizabeth	2	43°33′54.01″	70°11′54.26″	1.33±0.63
Kettle Cove, Cape Elizabeth	3	43°33′40.67″	70°13′04.99″	5.39±1.91
Nubble Light, York	4	43°09′56.78″	70°35′29.70″	0
**New Hampshire**				
Rye Beach, Rye	5	42°57′24.51″	70°46′40.23″	*P*
**Massachusetts**				
Back Beach, Rockport*	6	42°39′40.54″	70°37′23.98″	*P*
Good Harbor Beach, Gloucester*	7	42°37′11.31″	70°37′40.56″	*P*
Magnolia Beach, Gloucester*	8	42°34′28.95″	70°42′33.34″	*P*
Singing Beach, Manchester*	9	42°34′06.37″	70°45′39.85″	*P*
Stinky Beach, Manchester*	10	42°33′54.11″	70°47′11.18″	*P*
West Beach, Beverly	11	42°33′41.56″	70°48′15.80″	36.74±14.28
Castle Rock, Marblehead	12	42°29′58.67″	70°50′02.63″	18.80±2.56
Kings Beach, Swampscott	13	42°27′58.22″	70°55′15.64″	*P*
Canoe Beach, Nahant	14	42°25′10.09″	70°54′25.30″	28.41±6.11
Pumphouse Beach, Nahant	15	42°25′01.13″	70°54′25.27″	17.74±5.04
Pea Island, Nahant	16	42°24′54.33″	70°54′31.27″	79.3±3.81
Dorothy Cove, Nahant	17	42°25′14.31″	70°54′56.57″	*P*
Bay Shore Drive, Plymouth	18	41°56′59.02″	70°35′24.66″	*P*
Town Neck Beach, Sandwich	19	41°46′22.19″	70°29′30.42″	0
South Beach, Edgartown	20	41°21′05.98″	70°29′56.35″	*P*
**Rhode Island**				
Kings Beach, Newport	21	41°27′15.65″	71°20′35.70″	2.34±1.11
Fort Adams, Newport	22	41°28′36.60″	71°20′28.51″	13.50±4.13
Fort Wetherill, Jamestown	23	41°28′45.77″	71°21′40.69″	18.64±10.83
State Pier #5, Narragansett	24	41°25′20.17″	71°27′19.33″	0.65±0.65
Camp Cronin State Park, Narragansett	25	41°21′42.70″	71°29′18.99″	*P*
Southern Light, Block Island	26	40°09′04.73″	71°33′19.59″	*P*
Quonochontaug Pond, Charlestown	27	41°20′25.32″	71°43′12.04″	*P*
**New York**				
Latimer Reef, Southold	28	41°18′14.17″	71°55′42.78″	9.12±2.02
**Connecticut**				
Avery Point, Groton	29	41°18′54.15″	72°03′49.89″	0.36±0.36
Dock Road State Boat Ramp, Waterford	30	41°18′30.39″	72°08′54.59″	4.09±2.90
Two Tree Island, Waterford	31	41°17′38.50″	72°09′07.98″	23.10±4.88

*Notes*: Site numbers correspond to labels in Figure 1. *P* indicates locations where *Heterosiphonia* was observed drifting in the shallow subtidal but quantitative surveys were not conducted, and * indicates locations of weekly intertidal surveys.

### Statistical Analyses

Multivariate data were analyzed using Primer v. 6.0 (Primer-E Ltd., Plymouth, UK) to compare differences in subtidal community structure. Bray-Curtis similarity matrices were constructed on square-root transformed percent-cover data. We then ran a PERMANOVA (Permutational Multivariate Analysis of Variance; [19]) to determine if community compositions differed north and south of Cape Cod, Massachusetts. Univariate data were analyzed using JMP v. 9.0 (SAS Institute, Inc., Cary, North Carolina, USA). Regression was used to determine the relationship between species richness and abundance of *Heterosiphonia*. We conducted analyses of variance (ANOVAs) to assess spatial and temporal differences for intertidal drift surveys of *Heterosiphonia.* To assess temporal variability in our subtidal surveys we were only able to repeat subtidal surveys at four locations, two north (sites 14 & 15, Table 1) and two south (sites 21 & 22, Table 1). We were unable to satisfy the homogeneity of variances assumption for analyses of variances via transformation for these data [20]. Therefore, we used a generalized linear model (proc GENMOD in SAS v. 9.2, SAS Institute, Inc., Cary, North Carolina, USA) with a Poisson distribution and log link to assess temporal differences of *Heterosiphonia* abundance between early summer and late summer.

### Ethics Statement

No specific permits were required for the field surveys, as they were conducted from public access points or Northeastern University property (Marine Science Center, Nahant, Massachusetts, USA). The study did not involve any endangered or protected species or any protected locations.

## Results

**Figure 2:**
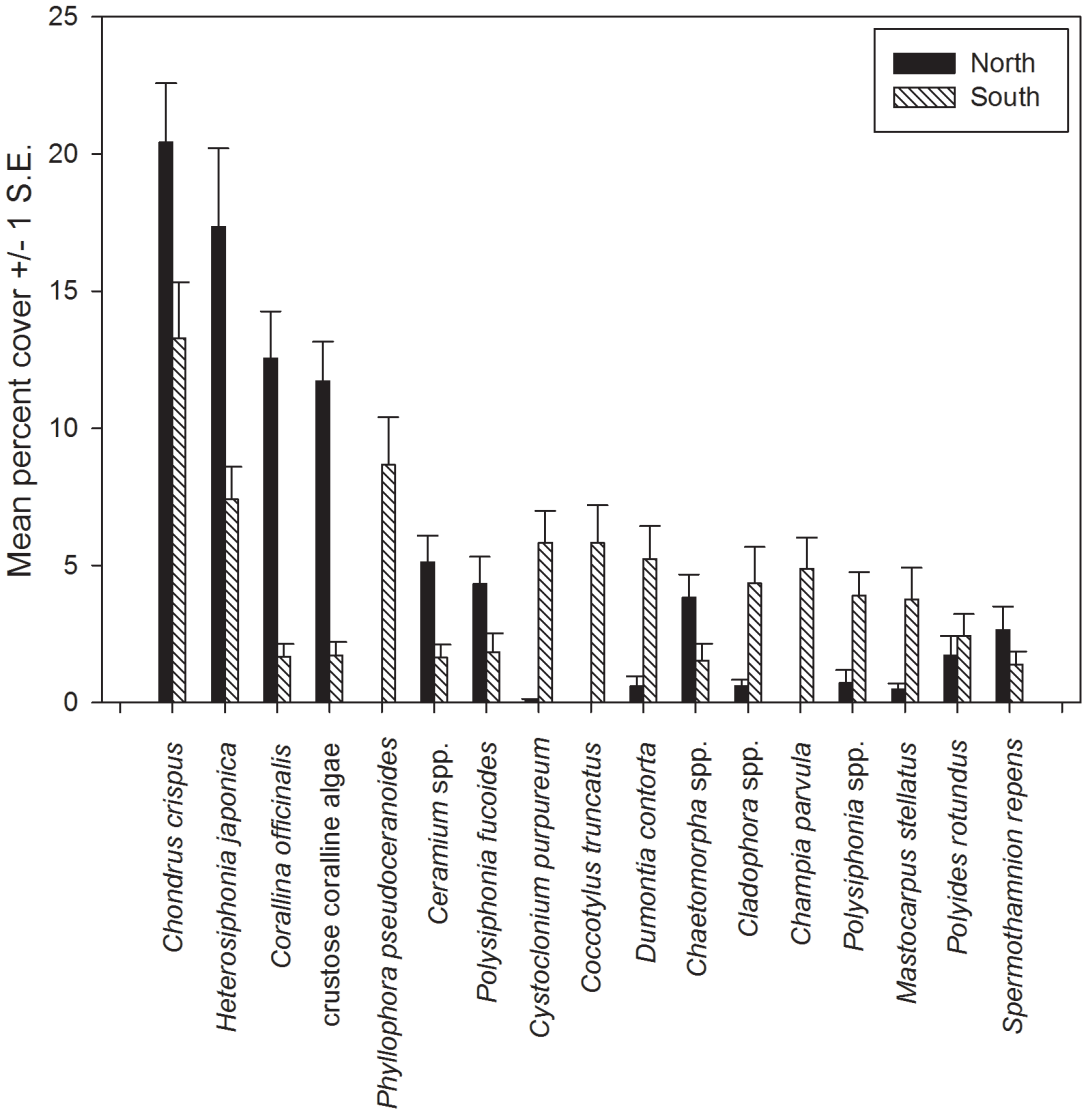
Relative abundances of sessile species (seaweeds and sessile invertebrates) in subtidal communities. North and south refer to the biogeographical barrier at Cape Cod, Massachusetts. Species listed comprised 80% of the overall community, and data are means ±1 At each site, a 20 m transect was haphazardly placed in the subtidal zone, at approximately the mid point of the species’ typical depth range (mean depth = 2.0±0.11 m). We placed a 0.0625 m^2^ quadrat every 2 m along the transect. Within each quadrat, the percent cover of each macroalgal and sessile invertebrate species was recorded. When present, a subsample of *Heterosiphonia* individuals was collected from each site. Upon collection, specimens were returned to the laboratory, where field identifications were confirmed under a compound microscope (100X) using characteristics from Schneider [8]. All individuals collected were either vegetative or tetrasporic. No fertile gametophytes were found during our sampling. At an additional 12 sites within our subtidal survey range, we examined the intertidal and/or shallow subtidal zone for evidence of drifting *Heterosiphonia* individuals. At each of these sites, individuals were collected and identified under a field microscope (40X) or laboratory microscope (100X). Due to logistical constraints, we clustered all crustose coralline algal species together, as *in situ* identifications to the species level proved impossible for this group. When possible, a subsample of *Heterosiphonia* individuals from most locations was pressed and deposited in the University of Rhode Island (KIRI) or Northeastern University (HNUB) herbarium collections.

Subtidal community composition differed significantly between the Acadian province and the Virginian province (p<0.001; Fig. 2). Northern subtidal communities were primarily composed of *Chondrus crispus, Heterosiphonia japonica*, crustose coralline algal species, and *Corallina officinalis*, which collectively comprised over 60% of the sessile species cover (means of 20%, 17%, 12% and 11%, respectively). While *Chondrus* and *Heterosiphonia* remained the most abundant species in southern subtidal communities (13% and 7% of the cover, respectively), *Phyllophora pseudoceranoides* also made up a substantial portion of the subtidal community (9%). There was also a greater diversity in community structure at southern sites, with 22 different species comprising 90% of the community. By contrast, only 13 species comprised 90% of the shallow subtidal community in the north (Fig. 2). Results were similar when *Heterosiphonia* was removed from the analysis (p<0.001).

**Figure 3:**
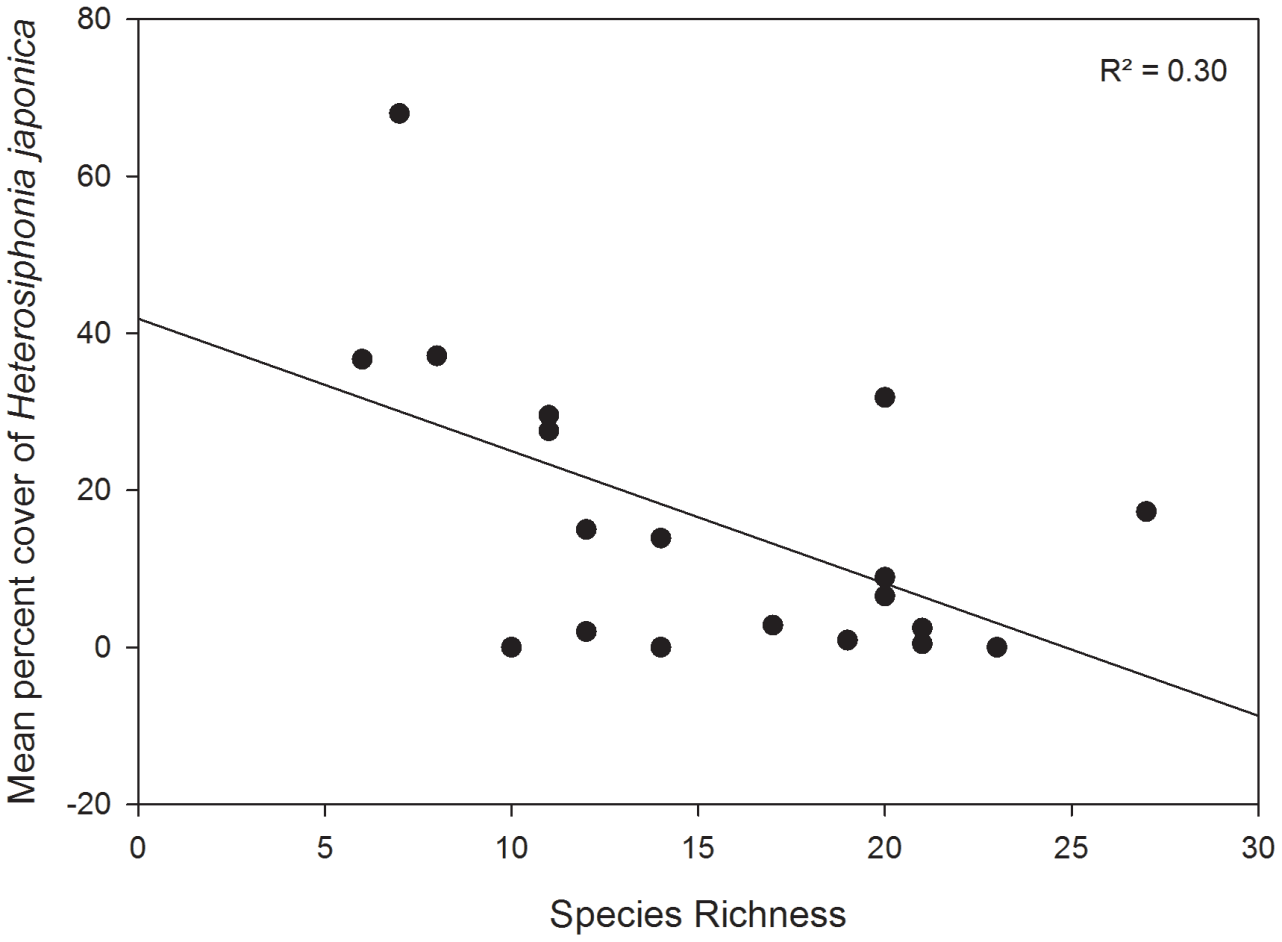
Relationship between sessile species richness and *Heterosiphonia japonica* abundance. Data presented are per plot, across all sites (regression; R^2^ = 0.30, p = 0.02).

Despite these differences in community compositions, *Heterosiphonia* was present at all but two of the subtidal sites surveyed (it was absent at Nubble Light, York, Maine and Town Neck Beach, Sandwich, Massachusetts) in both the Acadian province and the Virginian province. Relative abundances of *Heterosiphonia* ranged from 0.0 to 100 percent coverage to the north of Cape Cod (mean = 17.34±2.86%), while relative abundances were slightly lower south of Cape Cod (0.0% to 52.63%, mean = 7.41±1.19%; one-way ANOVA, F_1,183_ = 11.35, p<0.001). The average abundance of *Heterosiphonia* was lower in subtidal communities where species richness was higher (R^2^ = 0.30, p = 0.02; Fig. 3). During our surveys of intertidal wrack mats, *Heterosiphonia* comprised an average of 20% of biomass; however, abundances of up to 65% were seen on multiple occasions.

*Heterosiphonia* abundances also exhibited temporal and spatial variability, both subtidally and in intertidal wrack mats. Subtidal *Heterosiphonia* abundances were two orders of magnitude higher at sites surveyed during the early summer (May/June) than at the end of the summer (*X* ^2^ = 1676.52, p<0.001; Table 2). While intertidal wrack mat surveys were not conducted during the month of May, the abundance of *Heterosiphonia* was 40% higher during June than any of the other months surveyed (F_2,370_ = 9.34, p<0.001; Table 2). As expected, we found differences among sites surveyed (F_4,370_ = 15.95, p<0.001). There was also a significant site*week interaction for our intertidal drift surveys (p<0.001).

**Table 2:** Seasonality of *Heterosiphonia japonica* abundances.

	Subtidal(Percent Cover)		Intertidal Wrack(g/m^2^)
	North	South	
Early	43.80±7.38	7.65±2.35	17.47±9.33
Mid	–	–	8.01±3.13
Late	0.24±0.15	0.09±0.07	5.31±3.16

*Notes*: Within subtidal communities, *Heterosiphonia* abundance was at least two orders of magnitude higher in early summer (May/June) than during late summer (August; *X* ^2^ = 1676.52, p<0.001). At least twice as much *Heterosiphonia* biomass was found in intertidal wrack mats during June than in other months (F_6,45_ = 12.66, p<0.001).

## Discussion

While initial reports restricted the distribution of *Heterosiphonia* to Rhode Island [8], [9], this invader now occurs across a much larger biogeographic range. From this study alone, we have determined *Heterosiphonia* has become established in subtidal communities along >700 km of the western Atlantic coastline from Cape Elizabeth, Maine through Waterford, Connecticut. Furthermore, recent reports have documented the presence of *Heterosiphonia* in Atlantic Canada [9], and *Heterosiphonia* has been reported as far west as the mouth of the Connecticut River (J. Foertch, pers. comm.). This extensive range, with a continuous distribution spanning at least 700 km, became evident within only five years of the initial report of *Heterosiphonia* in Rhode Island in 2007 [9]. While we can not confirm how long *Heterosiphonia* may have been present (but unnoticed) in New England, if *Heterosiphonia* was indeed initially limited to sites in Rhode Island, as suggested by Schneider [8], then this incredibly rapid rate of expansion is comparable to the expansion throughout its invaded range along European coastlines; within 5 years of initial reports, the invader had spread >830 km along the Norwegian coast [10], [11]. Although the possibility of multiple introductions via shipping vectors throughout its western Atlantic range exists, it is also possible that *Heterosiphonia* successfully expanded its invaded range through rapid growth and nutrient uptake rates, high reproductive potential due to fragmentation, and release from natural herbivory (A. Drouin & N. Low, pers. comm.).

Based on the wide range of temperature and salinity tolerances of the European populations of *Heterosiphonia*, we believe this invader will continue to rapidly expand its geographic range along the western Atlantic coast, ultimately achieving a temperate to subtropical distribution and potentially invading locations from Florida to Newfoundland [16]. We are currently working to determine the lethal temperature and salinity ranges for the western Atlantic Ocean populations of *Heterosiphonia*. However, these populations are an exact genetic match to European populations [8], for which the thermal and salinity tolerances are known (0°C to 30°C, 10 to >30 psu; [16]). Thus, whereas this invader’s eventual range will likely be impacted by changes in temperature and salinity associated with climate change, a more pressing current concern is the likelihood that it will continue to expand its range rapidly, both northward and southward, to fill its thermal niche (i.e., its temperature and salinity tolerances allow it to grow well beyond its current geographic range).

*Heterosiphonia* was able to invade subtidal communities both north and south of Cape Cod, Massachusetts, a well-known biogeographical barrier. Despite the historical differences in both abiotic conditions and biological community structure between the two biogeographic provinces [17], [18], *Heterosiphonia* has become one of the most abundant macrophytes, on average, in these communities. However, the abundance of *Heterosiphonia* also appears to be spatially variable, with *Heterosiphonia* comprising up to 79% of total macrophyte cover at some locations, whereas in other communities where *Heterosiphonia* is present, it occupies <1% of the shallow subtidal community (Table 1). These data may be the result of our survey design, which was intended to rapidly assess the invader’s geographic range by maximizing the number of sites visited over the course of four months. As a result, we were not able to visit all sites on a regular basis.

However, we were able to capture some of the temporal variability in *Heterosiphonia* abundances in subtidal communities. During this study, four sites (two north and two south of Cape Cod, Massachusetts) were surveyed both at the beginning of the summer (May/June) and at the end of the summer (August). *Heterosiphonia* abundances were two orders of magnitude higher during May/June than during the end of the summer (Table 2). However, populations of *Heterosiphonia* appeared to be recovering following the end of this survey (September and October). Additionally, large populations of *Heterosiphonia* were present during the previous fall of 2011. Therefore, despite this decrease in abundance towards late summer, we postulate that *Heterosiphonia* populations may be experiencing a seasonal growth cycle, with extremely high abundances during the late spring (Table 2) and fall (late September – October). However, towards the middle of August, the abundance of *Heterosiphonia* was significantly reduced, even becoming undetectable in some locations where it was formerly abundant (Table 2). The seasonal pattern observed during 2012 is consistent with reproductive observations from European populations, where necrosis in cells of *Heterosiphonia* pseudolaterals was most prominently observed in late summer and fall [15], suggesting individuals were shedding small fragments. As fragmented pseudolaterals can function as vegetative propagules [15], the abscission of these structures during the late summer may lead to a secondary peak in the abundance of *Heterosiphonia* upon settlement and regrowth.

The temporal patterns observed in subtidal *Heterosiphonia* abundance paralleled patterns seen in intertidal wrack mats. Peak abundances of attached, subtidal *Heterosiphonia* were seen during May and began to decline in June (Table 2). In contrast, maximum intertidal wrack abundances did not begin to decline until July. This suggests that *Heterosiphonia* individuals became detached from the substratum during the late spring and early summer, existing primarily as drifting individuals. Indeed, many drifting specimens were observed during early summer months at various sites during our SCUBA surveys. The loss of these individuals from subtidal populations could further explain the decline in *Heterosiphonia* abundances seen during our surveys in late summer and provide further support for the likely importance of excised pseudolaterals as a means of reproduction for this invasive macrophyte, particularly in contributing to a second peak in abundance during early fall. However, it is currently unknown whether *Heterosiphonia* populations will maintain their high abundances through winter conditions (e.g. lower temperature, higher storm frequency and intensity).

Our surveys suggest that *Heterosiphonia* may have already altered subtidal community structure in areas it has invaded, as we observed lower seaweed species richness in communities characterized by greater *Heterosiphonia* abundance. These patterns are consistent with local extinction of native macroalgae due to competition with *Heterosiphonia*. It is also possible that communities with greater native macrophyte diversity are more resistant to invasion by *Heterosiphonia* [21], and we are currently conducting experiments to evaluate these possibilities.

Very little is currently known about the impacts of invasive seaweed species; ecological impacts have only been studied for ∼6% of the 277 known invasive seaweed species. Of these, only 6 studies have examined ecological impacts *in situ* [22], [23]. Collectively, our observations highlight community characteristics and population fluctuations across the current known range of a newly-discovered invasive macrophyte. The invasion and expansion of *Heterosiphonia* across multiple biogeographic provinces in the western North Atlantic Ocean provides an opportunity to understand the spread, impacts, and mechanisms associated with a marine invasion, providing critical information for management and amelioration of the impacts of this species and other marine invaders.
